# Intraoperative Analgesic Requirements in Dogs Receiving Retromammillary or Erector Spinae Plane Blocks for Thoracolumbar or Lumbar Hemilaminectomy: A Single-Center Retrospective Cohort Study

**DOI:** 10.3390/vetsci13090995

**Published:** 2026-09-20

**Authors:** Mariana Cavalcanti-McKenzie, Raiane A. Moura, Enzo Vettorato, Marta Romano, Ludovica Chiavaccini, Chiara De Gennaro, Elizabeth A. Maxwell, Pablo E. Otero, Diego A. Portela

**Affiliations:** 1 Department of Comparative, Diagnostic, and Population Medicine, College of Veterinary Medicine, University of Florida, Gainesville, FL 32608, USA; mmcken@midwestern.edu (M.C.-M.); mouraraiane@ufl.edu (R.A.M.); evettorato@ufl.edu (E.V.); marta.romano@ufl.edu (M.R.); lchiavaccini@ufl.edu (L.C.); cdegennaro@ufl.edu (C.D.G.); 2 Department of Specialty Medicine, College of Veterinary Medicine, Midwestern University, Glendale, AZ 85308, USA; 3 Department of Small Animal, Clinical Sciences, College of Veterinary Medicine, University of Florida, Gainesville, FL 32608, USA; emaxwell@ufl.edu; 4 Department of Anesthesiology and Pain Management, Faculdad de Ciencias Veterinarias, Universidad de Buenos Aires, Buenos Aires C1427CWN CABA, Argentina; potero@fvet.uba.ar; 5 Facultad Ciencias de la Vida, Escuela de Medicina Veterinaria, Universidad Andres Bello, Santiago 8370000, Chile

**Keywords:** canine, erector spinae plane block, hemilaminectomy, intraoperative rescue analgesia, regional anesthesia, retromammillary block

## Abstract

Thoracolumbar and lumbar hemilaminectomy in dogs requires effective perioperative analgesia. The erector spinae plane (ESP) and retromammillary (RM) blocks are ultrasound-guided regional anesthesia techniques targeting the dorsal branches of spinal nerves, but comparative clinical data are lacking. This retrospective cohort study compared perioperative analgesic requirements and complication rates between ESP (n = 62) and RM (n = 207) blocks in client-owned dogs undergoing hemilaminectomy. The primary outcome was intraoperative opioid rescue analgesia; secondary outcomes included rescue analgesia using any analgesic agent, perioperative opioid consumption (expressed as morphine equivalents, mg kg^−1^), pain scores, time to voluntary food intake, and complications. Multivariable logistic regression, with covariates selected using a directed acyclic graph, assessed independent predictors of intraoperative opioid rescue administration. Dogs receiving ESP blocks were more likely to require intraoperative opioid rescue than those receiving RM blocks (59.7% versus 38.7%; adjusted odds ratio (OR) 3.4, 95% confidence interval (CI) 1.7–6.6; *p* < 0.001). Rescue analgesia using any analgesic agent was also more frequent in the ESP group (64.5% versus 43.0%; *p* = 0.004). Postoperative pain scores, time to first voluntary food intake, and complications were similar between groups. The RM block was associated with reduced intraoperative rescue analgesia compared to the ESP block, though both techniques provided effective postoperative analgesia within multimodal protocols. Prospective randomized trials are warranted to confirm these findings.

## Simple Summary

Dogs undergoing spinal surgery for intervertebral disc disease require effective perioperative pain management. Regional anesthesia involves injecting a local anesthetic near nerves that transmit sensation from the surgical area and may reduce the need for additional analgesic drugs. This retrospective study compared two ultrasound-guided regional anesthesia techniques, the erector spinae plane (ESP) and the retromammillary (RM) block, in 269 dogs undergoing hemilaminectomy. Dogs receiving the RM block were less likely to require additional opioid or other analgesic drugs during surgery than dogs receiving the ESP block. Postoperative pain scores, time to voluntary food intake, and perioperative complication rates were similar between groups. These findings suggest that the RM block may provide more reliable intraoperative analgesia, whereas postoperative outcomes were generally comparable between techniques when each was incorporated into a multimodal analgesic protocol. Because this study used retrospectively collected clinical records, prospective randomized studies are needed to confirm these findings and determine whether one technique offers clinically meaningful advantages.

## 1. Introduction

The erector spinae plane (ESP) block is a fascial plane regional anesthesia technique that targets the medial and lateral branches of the dorsal branches of the spinal nerves (DBSN), which provide sensory innervation to the vertebral laminae, facet joints, and epaxial muscles [1,2,3,4]. In dogs undergoing hemilaminectomy, the ESP block has been shown to provide effective perioperative analgesia and reduce opioid requirements compared to systemic analgesic protocols [5,6,7].

Early descriptions of the ESP block in dogs closely followed the described human technique, using the transverse process as the landmark for local anesthetic injection into the erector spinae plane [1,8,9]. However, the unique anatomical features of the canine lumbar spine required modifications to optimize anesthetic spread to the DBSN. Consequently, the lumbar mammillary process was proposed as an alternative target landmark to the transverse process [10,11]. A subsequent cadaveric study demonstrated that ultrasound-guided ESP injections targeting the mammillary process, rather than the transverse process, resulted in superior staining of the medial branches of the DBSN when performed in the thoracolumbar and lumbar regions [3].

Recently, an anatomical study highlighted the close anatomical relationship between the mammillary processes of the caudal thoracic and lumbar vertebrae and the medial branches of the DBSN, leading to the development of a retromammillary (RM) approach [12]. This novel RM technique allows precise and reliable targeting of the medial branches of the DBSN and has been suggested as a suitable approach for dogs undergoing hemilaminectomy caudal to T11 [12].

At our institution, the RM and ESP blocks have been incorporated into a multimodal perioperative analgesic protocol for dogs undergoing thoracolumbar and lumbar hemilaminectomy surgery. Therefore, this retrospective cohort study was designed to compare the need for intraoperative rescue analgesia using opioids (primary outcome), followed by clinically relevant secondary outcomes (i.e., the requirement for intraoperative rescue analgesia using any analgesic agent, perioperative opioid consumption, pain scores, first voluntary food intake, and the incidence of perioperative complications) in dogs receiving either an RM or an ESP block as part of their perioperative pain management. We hypothesized that dogs receiving the RM block would have a lower requirement for intraoperative opioid rescue analgesia compared with those receiving the ESP block.

## 2. Materials and Methods

The Institutional Animal Care and Use Committee and the Hospital Research Review Committee of Veterinary Medicine, University of Florida, do not require an approved protocol for studies that use data collected from pre-existing medical records. Owner consent for the anonymous use of animals’ medical information for scientific and teaching purposes is obtained for all animals admitted to the University of Florida Veterinary Hospitals. Design and reporting of this retrospective cohort study followed the STROBE guidelines [13].

The University of Florida Small Animal Veterinary Hospital medical record database was searched for dogs that underwent thoracolumbar (T11–L1) and lumbar (L1–L7) hemilaminectomy between September 2020 and July 2024. Only dogs with complete anesthesia and postoperative treatment records were included in the study. Animals with incomplete medical records, with negative deep pain perception prior to surgery, or those that underwent bilateral hemilaminectomy were excluded from the data analysis. Treatment groups were assigned based on whether the animal received a unilateral ESP block (ESP group) or an RM block (RM group) as part of multimodal analgesic management.

### 2.1. Anesthetic and Analgesic Management

During the study period, dogs undergoing hemilaminectomy were managed according to the institutional standard of care described below. These general management principles were applied to all dogs; however, individual anesthetic and analgesic protocols were not standardized for the purposes of this retrospective study. Drug selection, dosing, regional anesthesia technique, intraoperative interventions, and postoperative analgesic adjustments were determined by the attending clinicians according to each dog’s clinical presentation, demeanor, perioperative pain score, American Society of Anesthesiologists physical status classification, comorbidities, and response to treatment.

All dogs typically received maropitant (1 mg kg^−1^) orally or intravenously (IV) within 24 h of the surgical procedure. As part of the preanesthetic medication protocol, a full µ-agonist opioid (methadone 0.1–0.4 mg kg^−1^ or hydromorphone 0.05–0.1 mg kg^−1^) alone, or in combination with dexmedetomidine (1–5 µg kg^−1^), was administered IV or intramuscularly (IM). General anesthesia was induced with propofol (2–5 mg kg^−1^, titrated to effect), alfaxalone (2–4 mg kg^−1^, titrated to effect), or a combination of propofol (titrated to effect) and ketamine (1–2 mg kg^−1^) or ketamine (5 mg kg^−1^) and midazolam (0.2–0.3 mg kg^−1^) IV. After induction and orotracheal intubation with an appropriately sized cuffed endotracheal tube, anesthesia was maintained with isoflurane using 100% oxygen as a carrier gas via a rebreathing system. The delivery fraction of inhalant was decided by the anesthetist based on clinical assessment of anesthesia depth and cardiovascular status. Intermittent positive-pressure ventilation was provided as needed to maintain end-tidal carbon dioxide tension between 35 and 45 mmHg. A dedicated anesthetist continuously monitored electrocardiography, end-tidal carbon dioxide tension, arterial blood pressure, peripheral oxygen saturation, and body temperature throughout the procedure, in accordance with the 2025 American College of Veterinary Anesthesia and Analgesia Small Animal Anesthesia and Sedation Monitoring Guidelines [14].

Intraoperative analgesia was provided by administration of an ultrasound-guided ESP or RM block, with or without the addition of IV full µ-agonist opioids and/or adjuvant analgesics (ketamine, lidocaine, and/or dexmedetomidine) as boluses or infusions. Ultrasound-guided ESP and RM blocks were performed as previously described, using the transverse process and the mammillary process as the respective anatomical landmarks [2,12]. The regional anesthetic technique, bupivacaine volume and concentration, and the use of adjuvants (e.g., dexmedetomidine) were selected at the attending anesthesiologist’s discretion. All dogs received the block ipsilateral to the planned hemilaminectomy.

Intraoperative rescue analgesia was administered at the anesthetist’s discretion, based on clinically relevant changes in heart rate, blood pressure, ventilatory asynchrony, and depth of anesthesia associated with surgical stimulation. After tracheal extubation, dogs were transferred to the intensive care unit or progressive care ward when body temperature was above 36.7 °C and clinical signs of pain or dysphoria were absent. Postoperative pain was scored using the Colorado State University canine acute pain scale every 2–4 h, performed by trained veterinary technicians.

Postoperative pain management consisted of IV administration of a full µ-agonist as a bolus or continuous rate infusion and oral adjuvant analgesics (e.g., gabapentin, non-steroidal anti-inflammatory drugs (NSAIDs)). Postoperative analgesic treatment was adjusted at the discretion of the clinician responsible for postoperative pain management. Food was offered 4 h after tracheal extubation and every 6 h thereafter.

### 2.2. Data Collection and Definition of Outcomes

Medical records were reviewed and the following information retrieved and compared between groups: demographic data, duration of anesthesia (defined as the interval from anesthetic induction to discontinuation of the inhalant anesthetic, including the time required for magnetic resonance imaging or computed tomography, when performed), duration of surgery, surgical site level, number of fenestrated laminae per surgery, and perioperative use of steroids or NSAIDs. Moreover, the dose and volume of bupivacaine used and whether additional dexmedetomidine was used as an adjuvant for ESP and RM blocks were recorded.

Preemptive analgesia was defined as the administration of any analgesic drug (i.e., opioids, ketamine, lidocaine, and/or dexmedetomidine) as part of the preanesthetic medication, induction, or at any time before and independently of surgical stimulation. Intraoperative opioid rescue analgesia (primary outcome) was defined as the administration of an opioid after the start of the surgical procedure. Intraoperative rescue analgesia using any analgesic agent was defined as the administration of an opioid and/or an adjuvant analgesic after the start of the surgical procedure. The proportion of dogs requiring any intraoperative rescue analgesia was compared between groups. Total intraoperative opioid consumption, defined as the sum of preemptive and intraoperative rescue opioid administration, was recorded and compared between groups. Cumulative doses of opioids administered from tracheal extubation to 48 h postoperatively were also recorded and compared. Because full µ-agonist opioids with different potencies were administered, direct comparison of cumulative doses was not possible. Opioid doses were therefore converted to morphine equivalents (ME) using the equivalence table published by the Hopkins Opioid Program, providing a common metric for comparison between groups, as previously described in veterinary studies [5,15]. For intraoperative opioid use, the cumulative dose was expressed as mg kg^−1^ of ME. Postoperative opioid consumption, expressed as cumulative mg kg^−1^ of ME, was compared at 24 and 48 h.

Pharmacological interventions used to stabilize the cardiovascular system intraoperatively, including intravenous crystalloid boluses, anticholinergic drugs (e.g., atropine or glycopyrrolate), and sympathomimetics (e.g., norepinephrine, dobutamine, dopamine, phenylephrine, or ephedrine), were compared between groups.

During recovery from general anesthesia, the time from inhalational anesthetic discontinuation to tracheal extubation, rectal temperature at tracheal extubation, and the occurrence of dysphoria were recorded and compared between groups. Over the 48 h following tracheal extubation, data collected included cumulative 24 and 48 h doses of analgesic drugs (opioids), postoperative pain scores, incidence of regurgitation, and time to first voluntary food intake, all of which were compared between groups. Additionally, any intraoperative or postoperative complications documented in the medical record, along with the drugs used to treat them, were documented.

### 2.3. Statistical Analysis

The sample size was calculated using an online sample size calculator (https://riskcalc.org/samplesize/, accessed on 19 August 2025). It was determined that 55% of dogs receiving an ESP block during hemilaminectomy required intraoperative opioid rescue analgesia [16]. Assuming an expected rescue rate of 35% in the RM group, a power (1–β) of 0.8, a significance level (α) of 0.05, and an enrollment ratio of 3:1 (RM:ESP), a total of 183 RM and 61 ESP cases were required. The unequal enrollment ratio was specified to reflect the anticipated distribution of cases at our institution, where the RM block largely replaced the ESP block for thoracolumbar and lumbar hemilaminectomies following publication of anatomical evidence supporting improved targeting of the medial branches of the DBSN with the RM technique [3,12]. Data were assessed for normality using the Shapiro–Wilk test. For continuous variables that did not meet normality assumptions but had sufficiently large sample sizes (n > 30 per group), a Welch’s *t*-test was applied based on the robustness of these methods under the Central Limit Theorem. Parametric data are reported as mean ± standard deviation (SD) with mean differences and 95% confidence intervals (CI). Nonparametric data were analyzed using the Mann–Whitney U test and are presented as median [interquartile range (IQR)] with the median difference and 95% CI. The median difference between groups was estimated using the Hodges-Lehmann estimator. Cumulative pain scores at 48 h were compared between groups. In addition, pain scores over time were summarized using the area under the curve (AUC) for each dog over the 48 h postoperative period. The AUC was calculated using the trapezoidal rule with a baseline set at Y = 0. Cumulative pain, expressed as AUC, was compared between groups using the Mann–Whitney U test. Categorical variables were compared using Fisher’s exact test or chi-square test, as appropriate.

Multivariable logistic regression was performed to assess the independent association between regional anesthesia technique (ESP versus RM) and the need for intraoperative opioid rescue analgesia. A directed acyclic graph (DAG) was constructed using DAGitty v3.1 (http://www.dagitty.net, accessed on 14 May 2026) to identify potential confounding pathways and guide covariate selection (Supplementary Figure S1). The minimal sufficient adjustment set determined by the DAG included premedication opioid dose (ME kg^−1^), preemptive opioid dose (ME kg^−1^), use of intraoperative adjuvant analgesics, number of laminae fenestrated, surgical duration (minutes), and patient weight (kg). Results are reported as adjusted odds ratios with 95% CI. Model fit was assessed using the Hosmer-Lemeshow goodness-of-fit test and the area under the receiver operating characteristic (ROC) curve. Multicollinearity was evaluated using variance inflation factors (VIF < 5 considered acceptable). Differences were considered significant when *p* < 0.05. Statistical analyses were performed using GraphPad Prism Version 8.0 (GraphPad Software Inc., La Jolla, CA, USA).

## 3. Results

A total of 346 medical records of dogs undergoing thoracolumbar or lumbar hemilaminectomy were retrieved, including 80 dogs that received the ESP block and 266 that received the RM block. After applying the exclusion criteria, 18 cases were excluded from the ESP group (12 due to negative deep pain perception and 6 due to incomplete medical records), and 59 cases were excluded from the RM group (51 due to negative deep pain perception and 8 for various reasons, including spinal fractures, bilateral surgical approach, or incomplete medical records). Consequently, 62 and 207 cases were analyzed in the ESP and RM groups, respectively.

Patient demographics (i.e., age, weight, sex, and breed size), surgical-related data (i.e., spinal level and number of laminae fenestrated), anesthetic duration, surgical duration, tracheal extubation time, and temperature at recovery are presented in Table 1. Perioperative steroids were administered to 63 of 207 dogs (30.4%) in the RM group and 20 of 62 dogs (32.2%) in the ESP group (*p* = 0.88). Postoperative NSAIDs were administered to 39 of 62 dogs (62.9%) in the ESP group and 93 of 207 dogs (44.9%) in the RM group (*p* = 0.014).

The bupivacaine dose used for the blocks was 2.1 ± 0.5 mg kg^−1^ in the ESP group and 2.2 ± 0.7 mg kg^−1^ in the RM group (mean diff. −0.1 mg kg^−1^, SE 0.1, 95% CI −0.3 to 0.1, *p* = 0.32). Dexmedetomidine (1 μg mL^−1^) was added as an adjuvant to the bupivacaine solution in 57.1% and 63.3% of the dogs in the ESP and RM groups, respectively (*p* = 0.38).

Dexmedetomidine was administered as part of the premedication protocol in 18 of 62 (28.6%) and 69 of 207 (33.3%) dogs in the ESP and RM groups, respectively (*p* = 0.54). Premedication with opioids (methadone or hydromorphone) was administered to 57 of 62 dogs (91.9%) in the ESP group and 173 of 207 dogs (83.6%) in the RM group (*p* = 0.15). In particular, methadone was administered in 51 of 62 (80.7%) of dogs in the ESP group and 167 of 207 (82.3%) of dogs in the RM group (*p* = 0.85); hydromorphone was administered in 6 of 62 (9.7%) in the ESP group and 6 of 207 (2.9%) in the RM group (*p* = 0.03). Among dogs receiving premedication with opioids, the mean ME dose was 0.41 ± 0.13 mg kg^−1^ in the ESP group and 0.44 ± 0.13 mg kg^−1^ in the RM group (mean diff. −0.02, SE 0.02, 95% CI −0.06–0.02, *p* = 0.24).

Intraoperative rescue analgesia with opioids was required more frequently in the ESP group than in the RM group (59.7% versus 38.7%; odds ratio (OR) 2.35, 95% confidence interval (CI) 1.29–4.27; *p* = 0.005). Among dogs receiving opioid rescue analgesia, however, the mean ME dose was lower in the ESP group (Table 2). Intraoperative rescue analgesia using any analgesic agent (i.e., opioids and/or adjuvant analgesics) was also required more frequently in the ESP group than in the RM group (64.5% versus 43.0%; OR 2.42, 95% CI 1.32–4.43; *p* = 0.004). Among dogs receiving rescue analgesia, 3 of 62 dogs (4.8%) in the ESP group and 9 of 207 dogs (4.3%) in the RM group received adjuvant analgesics alone without opioids. A higher proportion of dogs in the ESP group (69.4% versus 52.7%; OR 2.04, 95% CI 1.11–3.73; *p* = 0.028) received perioperative opioids, preemptive and/or rescue opioid administration, with correspondingly higher total intraoperative ME consumption (Table 2).

Intraoperative ketamine (as preemptive and/or rescue) was administered in 52 of 62 (83.9%) dogs in the ESP group and 179 of 207 (86.5%) in the RM group (*p* = 0.67), while lidocaine was administered in 4 of 62 (6.5%) and 12 of 207 (5.8%) dogs in the ESP and RM groups, respectively (*p* = 0.77).

After adjusting for premedication opioid dose, preemptive opioid dose, adjuvant analgesics, number of laminae fenestrated, patient weight, and surgical duration, dogs receiving the ESP block had significantly higher odds of requiring intraoperative opioid rescue analgesia compared to dogs receiving RM blocks (adjusted OR 3.4, 95% CI 1.7–6.6, *p* = 0.0002, Table 3). Longer surgical duration was independently associated with increased odds of opioid rescue analgesia (OR 1.01 per additional minute, 95% CI 1.001–1.017, *p* = 0.003), while higher preemptive opioid doses were associated with decreased odds of rescue (OR 0.56, 95% CI 0.29–0.93, *p* = 0.02). Patient weight showed a borderline association with rescue needs (OR 1.04 per kg, 95% CI 0.997–1.08, *p* = 0.07). Premedication opioid dose, adjuvant analgesics, and the number of laminae fenestrated were not significantly associated with opioid rescue analgesia in the multivariable model. The model demonstrated good fit (Hosmer-Lemeshow test *p* = 0.62) and moderate discrimination (area under the ROC curve 0.69, 95% CI 0.63–0.76). Variance inflation factor (VIF) values ranged from 1.03 to 1.33, indicating negligible multicollinearity among predictors.

There were no significant differences between groups in the need for pharmacological intervention to treat cardiovascular complications (i.e., use of anticholinergics, vasopressors, or fluid boluses), the incidence of regurgitation, or the presence of dysphoric recoveries (Table 4).

In the first 24 h postoperatively, 98.4% and 99.0% of dogs in the ESP and RM groups, respectively, received opioids (OR 1.68, 95% CI 0.11–14.61; *p* = 0.546). During the second postoperative day (24–48 h), 6.5% of dogs in the ESP group and 24.2% in the RM group received opioids (OR 4.62, 95% CI 1.65–12.37; *p* = 0.0018). The type of opioids administered in the postoperative period and the cumulative ME mg kg^−1^ administered in the first 24 and 48 h after surgery are shown in Table 5.

Median cumulative pain scores at 48 h were 0 (0–1) and 0 (0–2) in the ESP and RM groups, respectively (*p* = 0.18). The area under the pain score-time curve over 48 h was 0 (0–11) in the ESP group and 0 (0–13) in the RM group (median diff. 0, 95% CI 0–0, *p* = 0.35). Median time to first voluntary food intake was 21 (10–29) hours in the ESP group and 17 (9–28) hours in the RM group (*p* = 0.26). In the ESP and in the RM group, 2 of 62 dogs (3.2%) and 6 of 207 dogs (2.9%) did not eat during the 48 h postoperative evaluation period, respectively. Among dogs that ate during the evaluation period, the mean time to first meal was 21.8 ± 13.0 h in the ESP group and 20.2 ± 13.4 h in the RM group (mean diff. 1.66, SE 1.9, 95% CI −2.1–5.4, *p* = 0.39).

## 4. Discussion

This retrospective cohort study found that dogs undergoing thoracolumbar and lumbar hemilaminectomy and receiving the RM block as part of a multimodal analgesic protocol were less likely to require intraoperative opioid rescue analgesia than dogs receiving the ESP block. This association remained significant after adjustment for clinically relevant confounding variables using multivariable logistic regression, with dogs receiving the ESP block demonstrating over three times the odds of requiring opioid rescue analgesia compared to those receiving the RM block, although the retrospective study design precludes conclusions regarding causality. Postoperative pain scores and the incidence of perioperative complications were similar between groups. However, because the sample size of this current study was calculated based on intraoperative opioid rescue analgesia as the primary outcome, the study was specifically powered to detect a relevant difference in that variable. Consequently, all other inferential analyses should be interpreted with caution [17].

The ESP block was originally described using the transverse process of a thoracic vertebra as the landmark for injection [8]. This approach was subsequently extrapolated from the thoracic to the lumbar spine and applied in dogs undergoing thoracolumbar and lumbar hemilaminectomy, with the transverse process of a lumbar vertebra serving as the target landmark [2,18]. However, subsequent anatomical studies demonstrated that the medial branches of the DBSN are not closely associated with the lumbar transverse processes and suggested that the mammillary process may be a more appropriate landmark for lumbar injections [10]. Cadaveric investigations further demonstrated consistent staining of these nerve branches when RM injections were performed caudal to T11, whereas the conventional transverse process approach may result in less reliable spread to the intended neural targets [3,12]. In dogs, the anatomical relationship between the medial branches of the DBSN, the multifidus muscle, and the transverse processes varies along the vertebral column. Cranial to T11, the medial branches are located between the multifidus thoracis and the longissimus muscle; therefore, targeting the dorsolateral tip of a thoracic transverse process when performing an ESP block results in consistent involvement of the medial branches of the DBSN [2]. However, caudal to T11, the presence of mammillary processes alters the configuration of the multifidus muscle and the course of the medial branches of the DBSN. In this region, the medial branches lie in contact with the periosteum at the caudal aspect of the base of the mammillary process and are covered by the multifidus muscle [12]. Consequently, using the caudal aspect of the base of the mammillary process as the injection landmark caudal to T11 results in consistent staining of the medial branches of the DBSN. This anatomical feature could explain the lower incidence of intraoperative opioid rescue analgesia and the total rescue analgesia observed in dogs receiving the RM block and may reflect the more consistent blockade of the sensory innervation involved in hemilaminectomy surgery. Nevertheless, this proposed anatomical explanation remains speculative and cannot be confirmed from the clinical data collected in this retrospective study.

In a previous prospective study, approximately half of dogs undergoing lumbar ESP blocks required intraoperative rescue analgesia [6]. In the present study, dogs receiving the RM block, a technique also designed to target the medial branches of the DBSN, required rescue analgesia less frequently than those receiving ESP, supporting the potential of the RM block as an effective alternative to the lumbar ESP block. However, owing to the fundamental differences in study design, patient populations, and analgesic protocols between these studies, definitive conclusions regarding the superiority of the RM block over the lumbar ESP block using the mammillary process as a landmark cannot be drawn.

Despite the difference in intraoperative opioid rescue requirements, both regional anesthesia techniques appeared to provide effective postoperative analgesia. The majority of dogs in both groups experienced minimal postoperative pain, as evidenced by median cumulative pain scores of zero, the low area under the pain score-time curve values over the 48 h observation period, and the similar postoperative opioid consumption during the first 24 h.

A higher proportion of dogs in the RM group received opioids during the second postoperative day, resulting in greater cumulative opioid consumption at 48 h. Several factors may explain this observation. First, a higher proportion of dogs in the ESP group received NSAIDs postoperatively, which may have reduced postoperative opioid requirements in this group independent of the regional anesthesia technique. Second, dexmedetomidine added as an adjuvant to the bupivacaine solution may prolong the duration of sensory blockade; however, it was used in a similar proportion of dogs in both groups and is therefore unlikely to have systematically contributed to the observed between-group difference in intraoperative rescue analgesia requirements. Third, this difference may reflect variations in clinical judgment regarding opioid weaning between individual clinicians rather than true differences in postoperative analgesic requirements. Given the low median pain scores in both groups, the lack of standardized postoperative analgesic protocols, and the differences in NSAID administration, the clinical relevance of the observed difference in cumulative 48 h opioid consumption should be interpreted with caution.

The incidence of pharmacological interventions to treat cardiovascular complications, including administration of anticholinergics, vasopressors, or fluid boluses, was not different between groups. Among the interventions used to stabilize the cardiovascular system, anticholinergic administration was the most common treatment, suggesting that bradycardia may be the most frequent cardiovascular complication observed during this procedure [19]. However, owing to the retrospective nature of the study and the lack of a control group without a regional anesthesia technique, it is not possible to determine whether the inclusion of regional anesthesia had a positive or negative impact on cardiovascular stability in dogs undergoing hemilaminectomy surgery.

To account for potential confounding variables, multivariable logistic regression was used to assess the independent association between regional anesthesia technique and the need for intraoperative opioid rescue analgesia. Variables included in the model were selected a priori using a DAG to identify clinically relevant confounders and minimize bias. The use of a DAG to guide covariate selection represents a strength of this study, providing a systematic and transparent approach to identifying confounding pathways. Unlike automated variable selection methods (e.g., stepwise regression), which rely solely on statistical associations and may introduce bias, the DAG explicitly represents causal assumptions based on clinical knowledge and theoretical relationships between variables [20,21]. This approach ensures that relevant confounders are adjusted for while avoiding adjustment for mediators or colliders that could bias effect estimates. However, the DAG also makes explicit the limitations of this retrospective study by acknowledging unmeasured variables, including anesthetist experience, block quality, and individual patient pain sensitivity, which may influence both block selection and rescue analgesia requirements. While these unmeasured factors represent potential sources of residual confounding, the adjusted analysis confirmed that the association between block type and rescue analgesia persisted after controlling for premedication opioid dose, preemptive opioid dose, adjuvant analgesics, number of laminae fenestrated, patient weight, and surgical duration, strengthening the conclusion that the observed difference reflects a true effect of the regional technique rather than confounding by other perioperative factors.

Several limitations inherent to the retrospective study design must be acknowledged. First, rescue analgesia was defined as the administration of any analgesic drug after initiation of surgery, assuming that administration was associated with nociceptive stimulation. However, rescue analgesia may also have been influenced by factors unrelated to nociception, including timing of previous analgesic administration or changes in anesthetic depth. Therefore, the requirement for rescue analgesia may not accurately reflect the true intraoperative nociceptive state of the patient, particularly because no standardized criteria were used to determine when rescue analgesia should be administered. In addition, information regarding the specific surgical stimulus associated with rescue analgesia was not available from the medical records. Based on our clinical experience, nociceptive responses during hemilaminectomy in dogs receiving ESP or RM block most commonly occur during manipulation of the dorsal nerve root or extraction of disk material, structures that lie within the vertebral canal and are not directly targeted by either regional anesthesia technique used in this cohort. Therefore, the requirement for rescue analgesia may not exclusively reflect the efficacy of these techniques in desensitizing somatic nociception at the surgical site but may also be influenced by nociceptive input originating from tissues or neural structures beyond the intended distribution of the block. Second, the regional anesthesia technique was decided by the attending anesthesiologist, and it may have been influenced by factors such as individual preference, experience, or temporal trends in practice over the study period. Notably, the progressive replacement of the ESP block by the RM block during the study period represents a potential source of temporal bias, as changes in clinical practice or perioperative analgesic management protocols over time may have influenced the comparison between groups in ways that cannot be controlled in a retrospective design. The unequal group sizes reflect this real-world transition in our clinical practice and were anticipated in the study design, as reflected in the 3:1 enrollment ratio specified in the sample size calculation. Although multivariable regression was used to adjust for measured confounders, unmeasured confounding or selection bias cannot be entirely excluded. Third, pain assessment in the postoperative period relied on the Colorado State University acute pain scale, which is not a validated pain scale, and it may not capture all dimensions of pain experience. Fourth, multiple full µ-agonist opioids with different potencies were used, and opioid doses were converted to ME to standardize comparisons [5,15]. However, the reliability and accuracy of this method remain unknown in dogs. Finally, the study population was limited to a single institution, which may limit the generalizability of the findings to other settings or populations.

Despite these limitations, this study provides clinically relevant evidence supporting the use of the RM block as an effective alternative to the ESP block in dogs undergoing thoracolumbar and lumbar hemilaminectomy surgery. The reduced requirement for intraoperative and perioperative rescue analgesia and total rescue analgesia in dogs receiving the RM block, combined with comparable postoperative outcomes, suggests that the RM approach may offer advantages in terms of intraoperative analgesic efficacy. Future prospective, randomized studies are needed to confirm these findings and to further elucidate the mechanisms underlying the observed differences between techniques.

## 5. Conclusions

In dogs undergoing thoracolumbar and lumbar hemilaminectomy, the RM block was associated with a lower requirement for intraoperative opioid rescue analgesia compared to the ESP block, even after adjustment for clinically relevant confounding variables. Both regional anesthesia techniques provided effective postoperative analgesia as part of a multimodal analgesic protocol, with similar pain scores, time to first voluntary food intake, and incidence of perioperative complications. These findings suggest that the RM block may be associated with a lower requirement for intraoperative opioid rescue analgesia in this surgical population, although prospective randomized trials are needed to confirm these results.

## Figures and Tables

**Table 1:** Demographic distribution of dogs undergoing hemilaminectomy and administered either a retromammillary block (RM group) or an erector spinae plane (ESP) block (ESP group) as part of the perioperative analgesic management. Toy, small, medium, and large breeds correspond to body weights <6.9 kg, 7–12.9 kg, 1–25.9 kg, and >26 kg, respectively. The spinal level of hemilaminectomies is divided into thoracolumbar (T11–L1) and lumbar (L1–L7). Data are presented as mean ± standard deviation and number (percentage). The number of laminae fenestrated is presented as median (interquartile range, IQR).

	RM Group (n = 207)	Group ESP (n = 62)	*p* Value
Age (years)	5.4 ± 2.8	6.5 ± 3.0	0.015
Weight (kg)	11.1 ± 7.1	9.9 ± 6.1	0.19
Sex			0.36
Male	125 (60.4%)	30 (48.4%)	—
Female	82 (39.6%)	32 (51.6%)	—
Breed size			0.93
Toy (<6.9 kg)	63 (30.4%)	23 (37.1%)	—
Medium (7–12.9 kg)	90 (43.5%)	26 (41.9%)	—
Large (13.0–25.9 kg)	45 (21.7%)	11 (17.7%)	—
Giant (>26 kg)	9 (4.3%)	2 (3.2%)	—
Spinal level			0.77
Thoracolumbar (T11–L1)	108 (52.2%)	34 (54.8%)	—
Lumbar (L1–L7)	99 (47.8%)	28 (45.2%)	—
Number of laminae fenestrated	1 (1–2)	1 (1–2)	0.34
Duration of anesthesia (minutes)	266 ± 70	279 ± 79	0.21
Duration of surgery (minutes)	126 ± 39	132 ± 37	0.16
Tracheal extubation time (minutes)	9.8 ± 7.1	10.8 ± 7.9	0.39
Temperature at tracheal extubation °F (°C)	99.0 ± 2.1 (37.2 ± 1.17)	99.1 ± 1.5 (37.3 ± 0.83)	0.51

Significant difference, *p* < 0.05.

**Table 2:** Intraoperative opioid and rescue analgesia requirements in dogs undergoing hemilaminectomy surgery and receiving either a retromammillary block (RM group) or an erector spinae plane block (ESP group) as part of perioperative analgesic management. Opioid administration is expressed as morphine equivalents (ME, mg kg^−1^). Data for the entire cohort are presented as median (interquartile range, IQR). Data for dogs receiving opioids are presented as mean ± standard deviation (SD). Total intraoperative opioid consumption is the sum of preemptive and rescue opioid administration. Intraoperative opioid rescue analgesia is defined as administration of an opioid after the start of surgery. Total rescue analgesia is defined as the administration of any analgesic agent (opioids and/or adjuvant analgesics, including ketamine, lidocaine, and/or dexmedetomidine) after the start of surgery.

	RM Group (n = 207)	ESP Group (n = 62)	Difference (95% CI)	*p* Value
Total intraoperative opioids, n (%)	109 (52.7)	43 (69.4)	—	0.028
–ME requirements entire cohort:				
Median ME (IQR), mg kg^−1^	0.19 (0.00–0.40)	0.39 (0.00–0.49)	0.07 (0.00–0.20)	0.006
–ME in dogs receiving opioids:				
Mean ME ± SD, mg kg^−1^	0.61 ± 0.71	0.84 ± 1.10	0.23 (−0.13–0.59)	0.21
Intraoperative opioid rescue analgesia, n (%)	80 (38.7)	37 (59.7)	—	0.005
–Rescue dose entire cohort				
Median ME (IQR), mg kg^−1^	0.00 (0.00–0.32)	0.20 (0.00–0.40)	0.00 (0.00–0.10)	0.014
–Rescue dose in dogs receiving opioids:				
Mean ME ± SD, mg kg^−1^	0.55 ± 0.71	0.36 ± 0.17	−0.19 (−0.36–−0.02)	0.026
Total rescue analgesia (opioids/adjuvants), n (%)	89 (43.0)	40 (64.5)		0.004

CI, confidence interval; ME, morphine equivalent; RM, retromammillary block; ESP, erector spinae plane block. Significant difference, *p* < 0.05.

**Table 3:** Multivariable logistic regression analysis of factors associated with intraoperative opioid rescue analgesia in dogs undergoing thoracolumbar or lumbar hemilaminectomy (n = 269). Covariates were selected using a directed acyclic graph to identify potential confounders (Supplementary Figure S1).

Variable	Adjusted OR	95% CI	*p* Value
ESP block (versus RM)	3.37	1.76–6.66	0.0002
Premedication ME dose (mg kg^−1^)	0.39	0.10–1.46	0.16
Preemptive ME dose (mg kg^−1^)	0.56	0.29–0.93	0.02
Adjuvant analgesics	0.60	0.32–1.09	0.09
Number of spaces opened			
1 space	Reference	–	–
2 spaces vs. 1 space	1.46	0.8014–2.687	0.21
3 spaces vs. 1 space	1.142	0.3667–3.425	0.81
4 spaces vs. 1 space	1.41	0.12–32.75	0.78
Surgical duration (min^−1^)	1.00	1.00–1.01	0.003
Weight (kg)	1.04	0.99–1.08	0.07

OR, odds ratio; CI, confidence interval; ME, morphine equivalent; RM, retromammillary block; ESP, erector spinae plane block. Number of spaces was analyzed as a categorical variable, with 1 space as the reference category. Adjusted odds ratios and *p* values compare each category with the reference. Model diagnostics: Hosmer–Lemeshow statistic = 6.21, *p* = 0.624; area under the ROC curve = 0.69 (95% CI 0.63–0.76); VIF range, 1.03–1.33.

**Table 4:** Number (n) and percentage (%) of perioperative complications in dogs undergoing hemilaminectomy surgery and receiving either a retromammillary block (RM group) or an erector spinae plane block (ESP group) as part of the perioperative analgesic management.

Stage of Procedure	Treatment or Complication	Group		*p* Value
		RM n = 207	ESP n = 62	
Intraoperative				
	CV interventions	75 (36.2)	26 (41.9)	0.46
	Anticholinergics n (%)	52 (25.1)	18 (29.0)	0.62
	Sympathomimetics n (%)	7 (3.4)	4 (6.5)	0.28
	Fluid boluses n (%)	22 (10.6)	7 (11.3)	0.82
Postoperative				
	Dysphoria n (%)	48 (23.2)	16 (25.8)	0.67
	Regurgitation n (%)	27 (13.0)	8 (12.9)	>0.99
	Vomiting n (%)	0 (0%)	0 (0%)	–

CV: Cardiovascular intervention indicates dogs that received one or more treatments (anticholinergic, sympathomimetic, or fluid bolus) to stabilize intraoperative cardiovascular complications.

**Table 5:** Number (n) and percentage (%) of postoperative opioid consumption in dogs undergoing hemilaminectomy surgery and receiving either a retromammillary block (RM group) or an erector spinae plane block (ESP group) as part of the perioperative analgesic management.

	RM Group (n = 207)	ESP Group (n = 62)	Difference (95% CI)	*p* Value
Opioid Type Administered				
Methadone, n (%)	49 (23.7)	22 (35.5)	—	0.072
Fentanyl, n (%)	154 (74.4)	39 (62.9)	—	0.107
Hydromorphone, n (%)	2.0 (1.0)	0.0 (0.0)	—	>0.999
No opioids, n (%)	2.0 (1.0)	1.0 (1.6)	—	0.545
Cumulative ME Consumption				
-0–24 h postoperatively				
Mean ME ± SD, mg kg^−1^	4.7 ± 2.6	4.0 ± 2.8	−0.7 (−1.5–0.1)	0.078
-0–48 h postoperatively				
Mean ME ± SD, mg kg^−1^	5.6 ± 3.7	4.2 ± 2.9	−1.5 (−2.3–−0.6)	0.002

ME, morphine equivalent; SD, standard deviation; CI, confidence interval; RM group, dogs receiving the retromammillary block; ESP group, dogs receiving the erector spinae plane block. Significant difference, *p* < 0.05.

## Data Availability

The data presented in this study are openly available in Zenodo at 10.5281/zenodo.22692184 (uploaded on 19 September 2026).

## Supplementary Materials

The following supporting information can be downloaded at: https://www.mdpi.com/article/10.3390/vetsci13090995/s1, Figure S1 Directed acyclic graph.
