# Supplementary material for: Intraoperative Analgesic Requirements in Dogs Receiving Retromammillary or Erector Spinae Plane Blocks for Thoracolumbar or Lumbar Hemilaminectomy: A Single-Center Retrospective Cohort Study
Source: Vet Sci. 2026 Sep 20;13(9):995. doi: 10.3390/vetsci13090995 (PMC13611966; doi:10.3390/vetsci13090995)
Supplement: Supplementary file 1 [file vetsci-13-00995-s001.zip › vetsci-4543065-supplementary.pdf]

Supplementary file

Directed acyclic graph (DAG) depicting causal relationships between regional anesthesia technique and intraoperative rescue analgesia in dogs undergoing thoracolumbar or lumbar hemilaminectomy. The DAG was constructed using DAGitty v3.1 (http://www.dagitty.net) to identify potential confounding pathways and guide covariate selection for multivariable logistic regression analysis. The graph explicitly represents both measured and unmeasured variables that may influence the relationship between block type and rescue analgesia requirements.

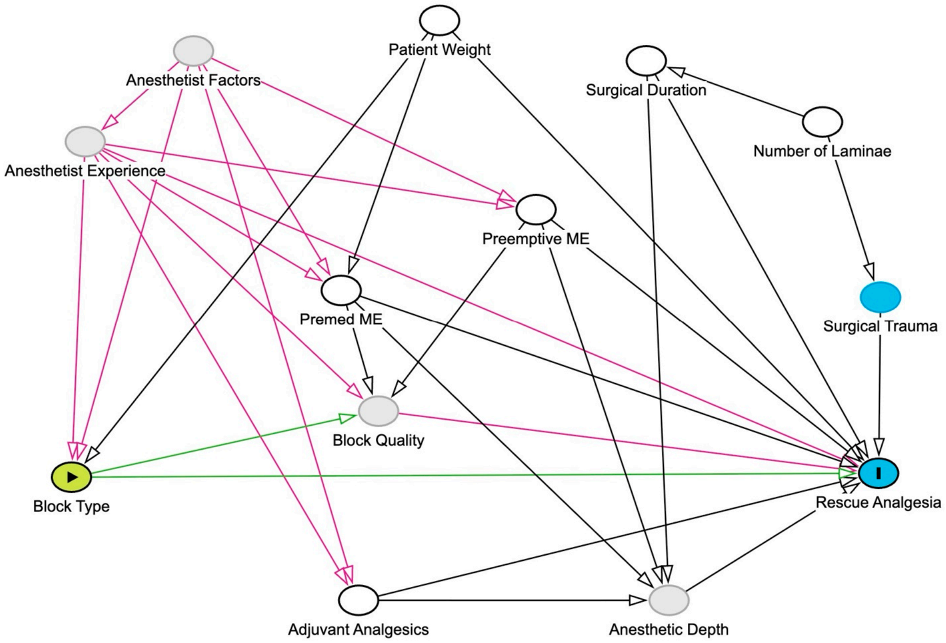

Figure S1. Node colors: The yellow node with the arrow symbol (▶) represents the exposure variable (Block Type: erector spinae plane [ESP] versus retromammillary [RM] block). The blue node with the black rim and the "I" symbol represents the outcome variable (Rescue Analgesia). White nodes represent measured covariates included in the multivariable regression model: Patient Weight, Premed ME (premedication morphine equivalents), Preemptive ME (preemptive morphine equivalents), Adjuvant Analgesics, Number of Laminae, and Surgical Duration. The light blue node represents a measured variable (Surgical Trauma) not adjusted for in the model because it lies on the causal pathway as a mediator between surgical factors and rescue analgesia. Gray nodes represent unmeasured latent variables (Anesthetist Experience, Anesthetist Factors, Block Quality, and Anesthetic Depth) that were not directly assessed but may contribute to confounding. Arrow colors: Green arrows represent the direct causal pathways of interest from Block Type to Rescue Analgesia, either directly or mediated through Block Quality. Pink arrows represent potential confounding pathways (backdoor paths) that connect the exposure and outcome through common causes and must be blocked by adjustment. Black arrows represent other causal relationships in the system that contribute to the overall causal structure.

The minimal sufficient adjustment set identified by DAGitty included Patient Weight, Premed ME, Preemptive ME, Adjuvant Analgesics, Number of Laminae, and Surgical Duration. These variables were included in the multivariable logistic regression model to estimate the unbiased causal effect of regional anesthesia technique on rescue analgesia requirements while blocking confounding pathways. Unmeasured variables are explicitly shown to acknowledge potential sources of residual confounding that could not be controlled for in this retrospective study. Surgical Trauma was not adjusted for to avoid blocking the causal pathway (mediator bias), as it represents an intermediate step between surgical factors and the outcome rather than a confounder.
